# Forces: A Motion Capture-Based Ergonomic Method for the Today’s World

**DOI:** 10.3390/s21155139

**Published:** 2021-07-29

**Authors:** Javier Marín, José J. Marín

**Affiliations:** 1 IDERGO (Research and Development in Ergonomics), I3A (Instituto de Investigación en Ingeniería de Aragón), University of Zaragoza, C/Mariano Esquillor s/n, 50018 Zaragoza, Spain; jjmarin@unizar.es; 2 Department of Design and Manufacturing Engineering, University of Zaragoza, C/Mariano Esquillor s/n, 50018 Zaragoza, Spain

**Keywords:** work-related musculoskeletal disorders, repetitive strain injuries, musculoskeletal risk assessment, industry 4.0, smart ergonomics, occupational healthcare, biomechanics, inertial measurement unit (IMU), kinematics and kinetics, design

## Abstract

Approximately three of every five workers are affected by musculoskeletal disorders, especially in production environments. In this regard, workstation ergonomic evaluations are especially beneficial for conducting preventive actions. Nevertheless, today’s context demonstrates that traditional ergonomic methods should lead to smart ergonomic methods. This document introduces the Forces ergonomic method, designed considering the possibilities of inertial motion capture technology and its applicability to evaluating actual workstations. This method calculates the joint risks for each posture and provides the total risk for the assessed workstation. In this calculation, Forces uses postural measurement and a kinetic estimation of all forces and torques that the joints support during movement. This paper details the method’s fundamentals to achieve structural validity, demonstrating that all parts that compose it are logical and well-founded. This method aims to aid prevention technicians in focusing on what matters: making decisions to improve workers’ health. Likewise, it aims to answer the current industry needs and reduce musculoskeletal disorders caused by repetitive tasks and lower the social, economic, and productivity losses that such disorders entail.

## 1. Introduction

Musculoskeletal disorders remain the most common work-related health problem, creating high costs for companies and affecting approximately three of every five workers [1]. Moreover, these injuries are prevalent in production environments where the worker must repeat tasks during the workday and are more frequent when the postural burden is higher [2]. Therefore, musculoskeletal disorders in the working population have significant repercussions on citizens’ quality of life and societal costs. Definitively, acting on prevention is acting on individual and collective quality of life.

Rotating workers among workstations, educating them about risk prevention, conducting permanent health surveillance, especially for more at-risk workers, and, if required, improving, redesigning, or correcting workstations that may cause more musculoskeletal injuries is paramount to prevent work-related musculoskeletal disorders [3]. In this regard, the ergonomic assessments of workstations are particularly beneficial to identify ergonomic issues that involve risk for the workers. Furthermore, this risk information is essential to take better corrective actions [4,5,6], so much so that, in many countries, regulations require conducting these assessments [7].

Typically, different observational methods or indices have long been applied to assess musculoskeletal disorder risk and conduct ergonomic evaluations. For repetitive work, Occupational Repetitive Action (OCRA, cited in the International Organization for Standardization (ISO) 11228-3) [8]; for load manipulation, the National Institute for Occupational Safety and Health (NIOSH) equation (cited in the ISO 11228-1) [9]; for posture load assessment, the method in ISO 11226 or other prestigious methods, such as the Rapid Entire Body Assessment (REBA) [10,11], Rapid Upper Limb Assessment (RULA) [12], or Ovako Working Analysis System (OWAS) [13]; finally, for more general purposes, such methods as the European Assembly Worksheet (EAWS) [14] or Postural Ergonomic Risk Assessment (PERA) [15]. These observational methods are useful in industrial environments because they do not require too much instrumentation—usually just a camera and a predefined template to take handwritten notes. With their widespread use, this ease of use has established these methods as reference methods in the industry [16].

However, the current context of Industry 4.0 [17] and interconnected intelligent devices in the Internet of things [18,19] highlights the need to update these observational methods [20,21]. Advances should also address risk prevention and consider the generalization and democratization of posturally healthy workstations. Therefore, we should strive for Ergonomics 4.0 or smart ergonomics processes [22,23,24,25]. Specifically, ergonomic methods should efficiently supply risk maps [26] integrated into this intelligent ecosystem. The risk maps are tables where the ergonomic risks of each assessed workstation can be identified, differentiating between body segments and where to delve into the detected problems and their origin to conduct improvement actions.

In this sense, several intrinsic factors related to observational methods make such advances necessary. First, the long time required to apply these methods becomes critical when ergonomic assessments must be conducted in large factories with many workstations [27]. In addition, the management of the information resulting from the evaluations is usually analyzed in a nonautomated manner, which makes it difficult for prevention technicians to focus on what matters: making decisions to improve their workers’ health. Finally, so much delay is sometimes accumulated in ergonomic evaluations that the proposed workstation corrections and modifications are received by the production area when they are no longer necessary because production conditions have already changed [27].

Additionally, the observational essence of these methods implies an inherent subjectivity. The movement analysis is usually based on the workstation observation with the naked eye, or at most, on the visualization of video [28]. Thus, direct and objective measurement techniques in productive workstations are promoted to address these limitations [20,21]. Technologies such as motion capture [27,29] or surface electromyography (EMG) [30] can be integrated into the ergonomic methodology to achieve an objective assessment.

Among these technologies, motion capture based on inertial measurement units (IMUs) presents relevant advantages. IMUs are electronic devices that capture movement through signal processing of the output data of various built-in sensors (accelerometers, gyroscopes, and magnetometers) [31,32,33]. IMU-based motion capture technology is a portable option because data collection can be conducted in a real environment while workers conduct productive operations. Likewise, it does not require excessive preparation time and is not excessively intrusive because it can be placed directly on the worker’s clothing, especially when compared to the EMG, which must adhere electrodes directly to the skin and requires highly qualified personnel [34,35].

Robert-Lachaine et al. [36] assessed the precision of an IMU-based motion capture system against an optoelectronic system as a reference (Optotrack) to be used as a tool for ergonomic evaluations (in situ occupational biomechanics) where there were varied and complex movements. They concluded that the measured errors remain acceptable during manual material-handling tasks, and the IMUs could track workers’ motions during their labor. However, this technology is not without limitations; the built-in magnetometers are highly sensitive to disturbances in the Earth’s magnetic field, especially in indoor environments, and a drift artifact is caused by cumulative gyro integration error [37].

In addition to the applicability of direct measurement in the industrial environment concerning the technology used, it is essential to consider the information analysis method that the specific technology can provide. Regarding this, Caputo et al. [29] applied the EAWS [14] method to production lines by replacing posture observation conducted by an assessor with IMU-based motion capture. Likewise, Peppoloni et al. [30] and Vignals et al. [38] used a similar process, applying the RULA method to a laboratory simulated environment. This same idea is the one observed in commercial software, such as ViveLab Ergo [39], NAWO Live [40], or ScaleFit [41], that allows the application of ergonomic observational methods using motion capture to automate the postural load analysis.

Regarding these examples, we highlight the need to develop and design ergonomic methods conceived considering the technology on which they are based. The technology provides a large amount of precise and objective information that should benefit the analysis and foster the automation of the entire ergonomic evaluation process. For example, it makes little sense to rate the postural load by steps and not by interpolation if the measured angles of the joints are continuous. Along these lines, Zare et al. [27] proposed a novel score based on motion capture, including the postural factor as a first approximation toward the assessment. This study leads us to highlight another point: the need to consider and objectify other ergonomic factors, such as the efforts or accelerations that occur in the body, in addition to the postural load. An example of this is the study by Peppoloni et al. [30], who addressed this need using simultaneous IMU and EMG measurements to study both postures and the efforts made.

To address the described challenges, we propose the Forces ergonomic method designed considering the possibilities offered by IMU motion capture for application in industrial environments to record workers’ movements at their workstations. This method calculates the joint risks for each posture and provides a total risk for the assessed workstation. In this calculation, Forces uses postural measurement, a kinetic estimation of all forces and torques that the joints support derived from the movement, and the external forces exerted by the worker, hence its name. In this paper, the method’s fundamentals are detailed to achieve structural validity, ensuring that all parts that compose it are logical and well-founded. This method is expected to address the current needs of Ergonomics 4.0, reducing work-related musculoskeletal disorders derived from repetitive tasks, and minimizing the social, economic, and productivity losses that these injuries entail.

## 2. Materials and Methods

The Forces method aims to estimate the risk of musculoskeletal disorders in repetitive workstations organized in work cycles. This estimation requires measuring the work cycle with motion capture in a workstation with an operator and determining certain information associated with the evaluated work. Then, the Forces scoring system determines the risk of suffering musculoskeletal disorders in each assessed joint, specifically in the lumbar and cervical spine, shoulders, elbows, wrists, and knees.

The Forces method was developed by a team of engineers from different specialties (biomedical, industrial, mechanical, and product design) through an iterative process of “research-action” [42]. It resulted from collaboration through different research projects with prevention technicians and health practitioners involved in ergonomics and health surveillance of industrial and prevention companies. In these projects, numerous ergonomic evaluations with observational methods and varied IMU-based motion captures in manufacturing environments were accomplished. Some of these studies led to the doctoral thesis by Boné [43] and laid the conceptual foundations for the Forces method. All this work evolved Forces until it reached the current version presented here.

As mentioned, this research aims to verify and demonstrate the method’s usefulness to fulfill its stated purpose effectively and efficiently [44]. Under this statement, the usefulness of the ergonomic Forces method is associated with a double condition: that (1) the method provides the risks correctly for effectiveness, which implies (2) adequate functionality and applicability, overcoming the barriers described in the introduction for efficiency [45].

To demonstrate both conditions, we rely on the “theoretical structural validity” described by Pedersen [45], which assumes that the parts that compose the new methodology are validated and supported by the bibliography and that the joint of these parts is consistent and defines a flow of information without redundancies or errors. This concept coincides with the “face validity” described by Takala et al. [46], which implies the method has solid foundations and correct data collection and analysis processes.

From this perspective, this section presents the measurement and analysis fundamentals to provide consistent and structurally valid risk values and describes the experimentation conducted to define the maximum effort thresholds to compute the risk values (effectiveness). Subsequently, in Section 4, the applicability of this method is discussed through a use case (efficiency). Finally, this article is accompanied by Supplementary Materials (Forces_Tables.xlsx), which is cited throughout this section because it contains tables and graphics for the Forces application.

### 2.1. Motion Capture System

Concerning motion analysis, the Forces method does not require the use of a specific motion capture system. In our case, we used the MoveHuman (MH) Sensor motion capture system developed by the IDERGO Research Group, with the system’s MH-IMU configuration, which was recently described by Marín et al. [35,47]. This system is based on next-generation IMU (NGIMU) devices developed by X-io technologies [48], which measure the rotations via signal processing in embedded sensors (accelerometers, gyroscopes, and magnetometers) and transmit the information via Wi-Fi. Specifically, we use the full-body setting (15 IMUs) configured at 60 Hz and placed on the body with elastic bands, exactly as Marín et al. [35] explained. The calculation process of the Forces method was implemented on the MH application under the same development platform: Vizard (6.2 version, WorldViz, Santa Barbara, CA, USA, 2020), which is based on Python.

**Figure 1:**
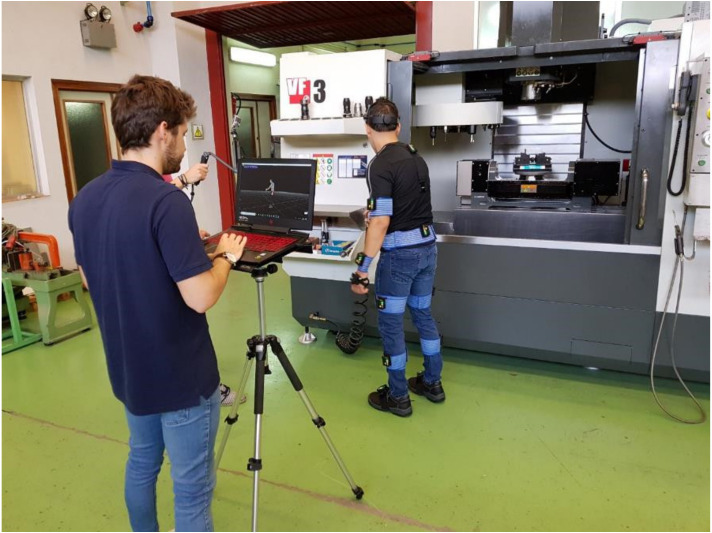

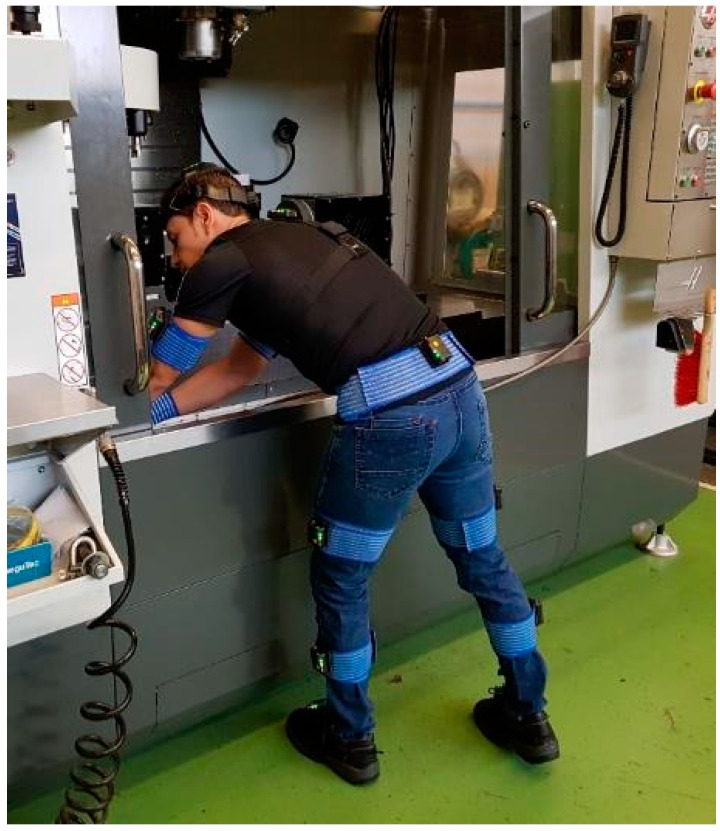
Motion capture in the field, placement of sensors on the worker’s clothing.

A laptop, which can be placed on a tripod tray, a portable router, and two video cameras to take pictures of workers’ movements are required to use the MH system in the field (Figure 1). Cameras are not required for motion capture; they only offer additional visual information. One of the cameras is directly connected to the computer and provides an overview of the workstation (Logitech C920 webcam), and the other is remotely controlled and managed by an assessor to obtain details of the worker’s hand actions (sports camera GITUP GIT2). The captured motion, along with the video images, allows for ergonomic assessment.

The use of the MH system is justified in the first place for its reproducibility. In a gait analysis experiment with healthy subjects, it reached an average intraclass correlation coefficient of 0.90 in the representative variables [35]. Reproducibility is the most general and important indicator to ensure the proper functioning of motion capture systems [49,50]. High reproducibility ensures that the system produces similar data under the same conditions and has sufficient precision to compare results. Additionally, the MH system incorporates an anatomical calibration procedure or sensor-to-segment alignment [51,52,53] called Fitbody. This process is applied for a few seconds and is effectively transmitted to the participant. The worker adopts a specific neutral body position (posture illustrated in Figure 5a of the paper by Marín et al. [35]), and then the operator of the capture system executes the Fitbody function, ensuring that the participant’s position is adequate [35,47].

This Fitbody process allows for the deactivation of the IMUs’ magnetometers to avoid the magnetic influence. These magnetic disturbances are expected in industrial environments, where there is equipment, wiring, and electromagnetic signals. However, disabling the magnetometers is not without limitations; due to the internal IMU sensor fusion algorithm, there is a drift error that increases with time in the absence of magnetic information; thus, the calibration duration depends on the integration of the drift of the gyros. For this reason, we apply the Marín et al. [35] approach; they demonstrated that if captures are limited to about 3 min, which is longer than the usual manufacturing cycles, and a Ftibody calibration process is accomplished before each recording, a satisfactory reproducibility is achieved. However, since this approach has limitations, other options to prolong the capture time are included in the discussion section.

### 2.2. Human Model, Worker Anthropometry, and Percentiles

**Figure 2:**
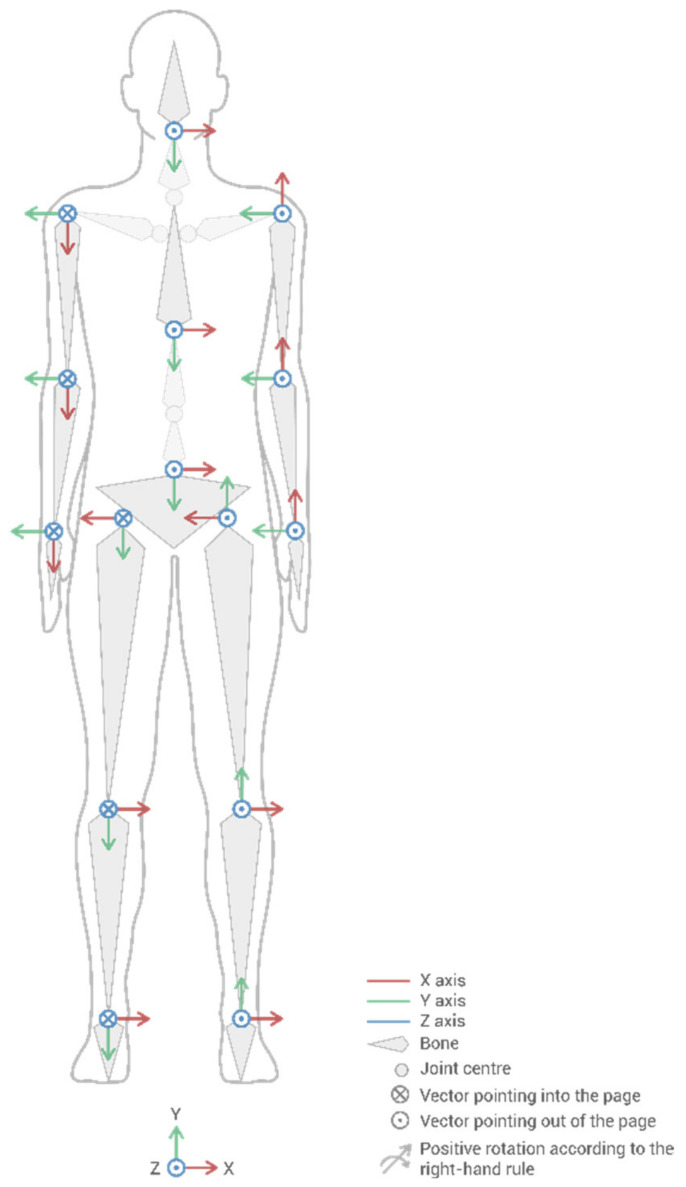
Human model in a neutral position. Sign convention is established to interpret the bone rotation directions according to the right-hand rule [35].

Figure 2 depicts the human model embedded in the MH system. It illustrates the neutral position (zero rotations) and the local coordinate system for each bone. The bone’s coordinate systems are situated on the centers of the joints at the beginning of each bone (e.g., the center of the femur bone is situated on the center of the hip joint), except for the pelvic bone, where the center is located at the geometric center of the pelvis. This convention facilitates the interpretation of the rotations for each joint, which follow the right-hand rule.

The dimensions of different body segments of the human model directly influence kinematic and kinetic parameters and, therefore, the risk assessment. The system requires the assessor to enter the height of the worker and the distance between the elbows (olecranon) in a neutral position to adjust the human model in Figure 2 to the worker’s anthropometry. This distance is projected on the anatomical frontal plane and allows adapting the model’s shoulder width internally. In this manner, the system generates a human model whose parameters are obtained by interpolation from the Huston [54] male and female models, included in Table 1. However, it is possible to recalculate the Forces method a posteriori, considering other anthropometric characteristics (i.e., scaling the human model to the P05, P50, or P95 percentiles of male or female).

In the same vein, the weight considered for the human model is calculated according to Table 1, interpolated with the worker’s height. In this regard, the worker’s weight can be introduced if we aim to estimate risk for a particular operator. However, if we intend to obtain the risk for the workstation, it is preferable to use the weight associated with the anthropometric data in Table 1.

Likewise, for kinetic calculations, Table 2 lists the percentage assigned to each body segment of the total body weight (%*W*) and the position of the center of gravity of each segment as a percentage of the length of the bone concerning its origin from which it rotates (%*CG*; see origins in the human model in Figure 2). Note that variables along the paper are included in italics to further identify them. The data in Table 2 were obtained from Huston’s book [54] and transformed into the coordinate system of the MH system. 

Finally, Table 2 also includes the inertial tensors of each male and female body segment (*T*) that Huston [54] considered and their percentiles. Inertial tensors indicate the inertia of the body segment in each of the axes of rotation and are necessary for calculating the inertia forces caused by the movement applied in the centers of gravity of each segment. The tensors correspond to 3 × 3 diagonal matrices; therefore, only the diagonal values of each tensor are included. The first value is the moment of inertia for the *x*-axis, the second for the *y*-axis, and the third for the *z*-axis; the *xyz* axes correspond to the global coordinate system at the bottom of Figure 1. Interpolating between percentiles using the weight of the human model is necessary to calculate the intermediate values.

### 2.3. Kinematics: Calculating Rotations, Displacements, Velocities, and Accelerations in Joints

After describing the capture system and human model, the complete kinematics must be calculated to apply the Forces method. This application requires computing the joint rotation, displacement, velocity, and angular and linear acceleration for each capture instant. Marín et al. [35] described how to calculate the kinematics with the MH capture system. For this, the rotations measured by the sensors are transformed to relative joint rotations around each of the axes (*Rx*, *Ry*, *Rz*). The described right-hand rule and coordinate systems of Figure 1 must be considered to interpret these rotations as positive or negative. Likewise, as Marín et al. [35] explained, this model provides the displacement of each bone’s center along each axis. These displacements are calculated by direct kinematics [55] using the joint rotations and lengths of each bone [55,56].

Each joint’s angular and linear velocity and acceleration can be calculated from the rotation and displacement. Angular velocity is the first derivative of the *Rx*, *Ry*, *Rz* curves, and the angular acceleration is the second derivative (calculated using Python’s Scipy module). Moreover, the linear velocity and acceleration can be calculated in the same manner from the displacement. Therefore, from the motion capture, the complete kinematics values of the evaluated worker are known, which is necessary for the next stage of the process.

### 2.4. Kinetics: Calculation of Forces and Torques in Joints

The next step is to calculate the kinetics in each joint (i.e., the module and direction of the force and torque vectors). Various authors have described how to calculate the forces and torques in each joint from the kinematics [57,58,59,60]. In our case, the MH application calculates the forces and torques in each joint and at each instance using the Huston [54] equations, modeling the human body as a set of bars with a known length, weight, center of gravity, and tensor of inertia (described in Section 2.2). These bars are linked by ball joints and move in space, considering that the body is in equilibrium at all instances.

**Figure 3:**
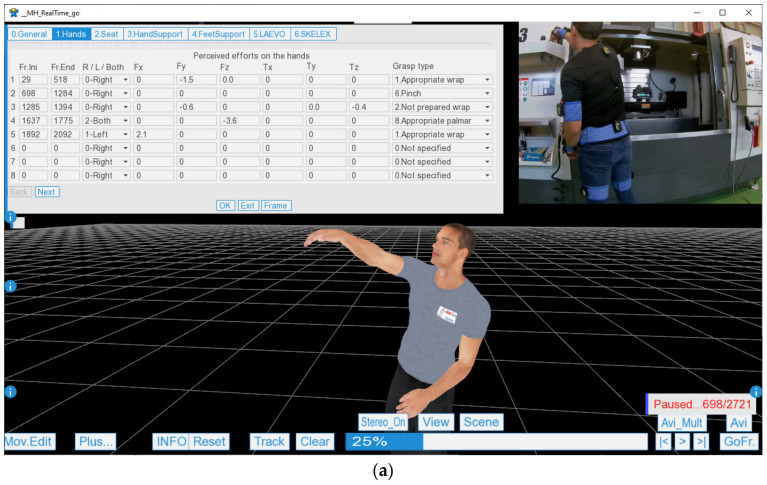

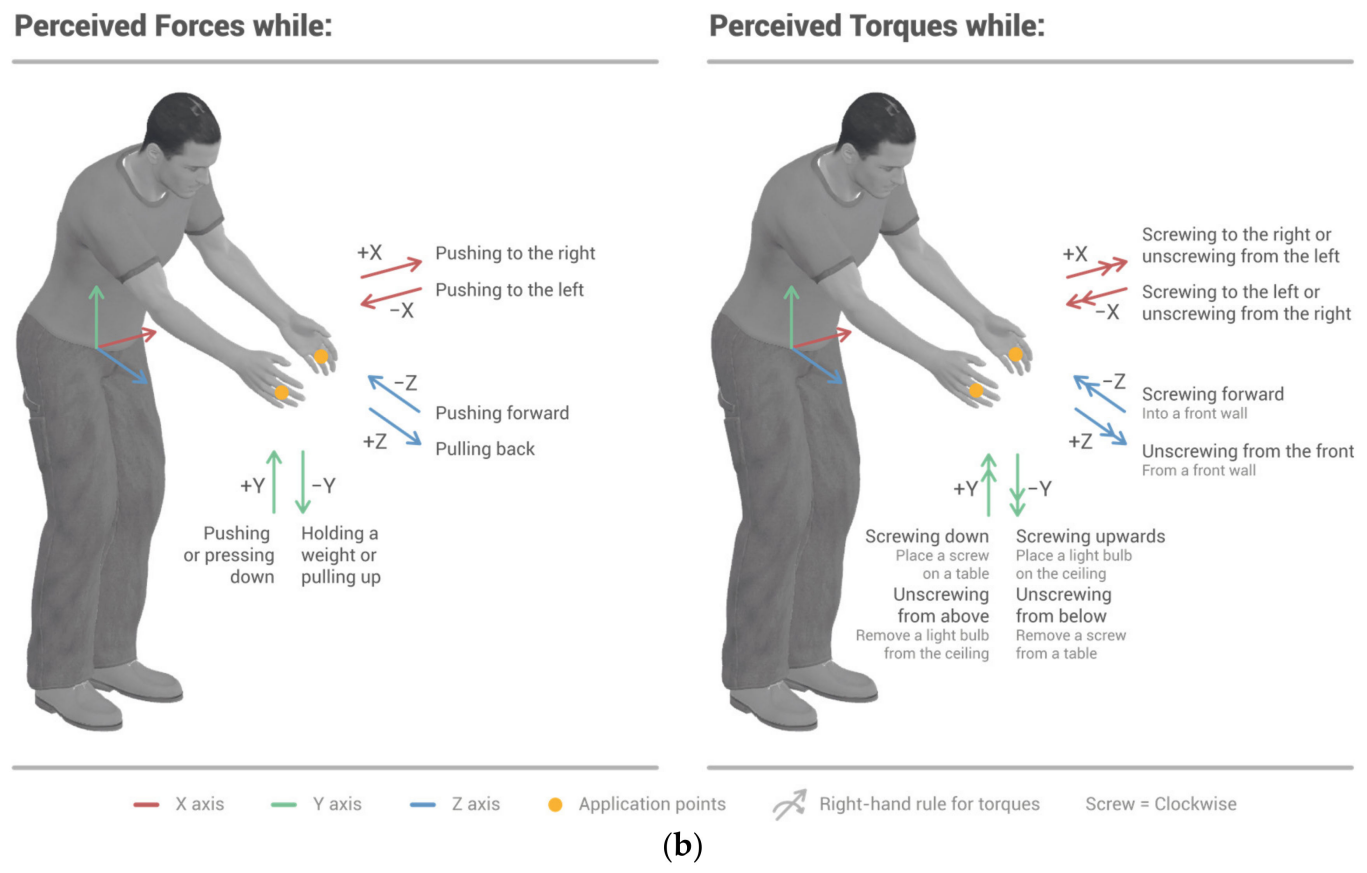
(**a**) Form to introduce external forces and torques exerted with the hands. (**b**) Criteria and examples of interpreting force and torque vectors are entered in the form.

However, before solving the Huston equations [54], the forces and external torques regarding the workers’ performance with the hands to accomplish actions during the work cycle must be known. For this, the form in Figure 3a was implemented. In this form, each row represents an action performed by the worker. It is required to indicate the range of frames during the action (Fr.Ini, Fr.End), whether the action is exerted with one or both hands (right (R), left (L), or both), the force vector (*Fx*, *Fy*, *Fz*), the torque (*Tx*, *Ty*, *Tz*), and, finally, the grasp type.

The coordinate system linked to the system of the pelvic bone was established to facilitate the entry of the force and torque vectors, as illustrated in Figure 3b. This coordinate system rotates with the pelvis, but the *y*-axis was established vertically to the ground (i.e., it rotates with the pelvis), and the *z-* and *x*-axes are parallel to the ground. The vector sign (positive or negative) is interpreted as the perceived force or torque in the hands due to the action. Figure 3b includes examples of forces and torques and their interpretation for data entry into the form. In addition, if the force or torque is not exerted in an orthogonal direction to the *xyz* described coordinate, it is possible to make a decomposition by projecting the vector in these three axes.

Once the hand actions are introduced, it is necessary to determine the support points of the body at each moment. If the support situation is standing or walking, the algorithm that incorporates the MH application detects whether one or both feet are supported [35]. In these cases, it is not necessary to enter additional information regarding the support. However, if the worker is seated or has one or both hands supported, it is necessary to enter the range of frames where these situations occur. For this purpose, a form similar to the one in Figure 3a is available.

**Figure 4:**
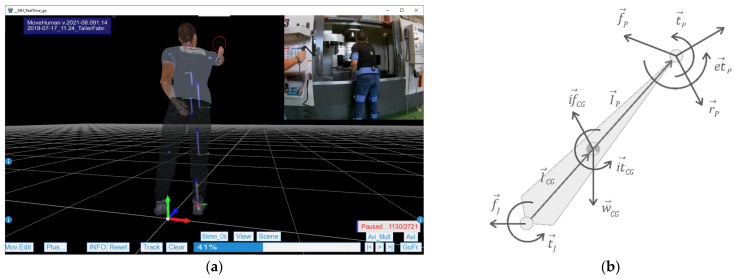
Kinetics calculation. (**a**) Result of the kinetic calculation marking the support situations (red points), the forces (blue arrows), and torques (green arrows) on the joints. (**b**) Segment isolation to calculate force and torque vectors supported by a specific joint.

With these considerations, physics and biomechanics equations are applied to obtain the forces in each joint. The calculation is conducted posture by posture, following the sequence of captured frames, and with the condition that the sum of the forces and torques in the three axes of space must always be zero. Figure 4 illustrates the result in an example posture, where the force vectors are represented in blue, the torque vectors are in green, and the support is indicated with a red point.

The calculations are organized in two stages. The first stage considers the body as a whole and estimates the reactions at the support points. The following factors must be considered for the estimation: (1) position of the center of gravity of each body segment; (2) weight of each body segment applied in each of these centers; (3) sense and magnitude of the external efforts of the worker applied on the center of gravity of the hand; (4) inertial forces derived from the linear and angular acceleration of the movement applied on the centers of gravity of each body segment (in the opposite direction of the acceleration vector); and (5) support points of the body (standing, sitting, or with one or both hands in support).

In the second stage, the internal stresses in the joints (i.e., the force and torque vectors that each body segment is exerting on the joint that allows it to rotate) are calculated. In this manner, the efforts of the shoulder are exerted by the arm and the following kinematic chain (forearm and hand) in each frame. Thus, the calculation begins with the distal joints, wrists, and ankles and continues into the body, following the kinematic chain until reaching the pelvis, which corresponds to the origin segment of the body for both the upper and lower limbs.

Following this structure, Equation (1) shows how to calculate the vector ${\vec{f}}_{J}$, representing the force supported by a joint. To perform this calculation at a specific joint, the anterior segment of the kinematic chain is isolated, as shown in Figure 4b. In this manner, if we calculate ${\vec{f}}_{J}$, for example, at the wrist joint, the segment to be isolated is the hand and, for example, at the elbow joint, the segment to be isolated is the forearm. In Equation (1), the vector ${\vec{w}}_{CG}$ is the self-weight of the segment, which points continuously towards the ground; ${\vec{if}}_{CG}$ is the inertial force, which depends on the linear acceleration and the mass of the segment; ${\vec{f}}_{P}$ is the force vector in the previous joint, calculated following this same process; ${\vec{ef}}_{P}$ is the external force applied on the previous joint, which can be entered in the form described in Figure 3; finally, ${\vec{r}}_{P}$ is the reaction in the previous join, calculated in the described first stage, according to the support conditions.

(1) $${\vec{f}}_{J}={\vec{w}}_{CG}+{\vec{if}}_{CG}+{\vec{f}}_{P}+{\vec{ef}}_{P}+{\vec{r}}_{P}.$$

(2) $${\vec{t}}_{J}={\vec{l}}_{CG}\times \left({\vec{w}}_{CG}+{\vec{if}}_{CG}\right)+{\vec{l}}_{P}\times \left({\vec{f}}_{P}+{\vec{ef}}_{P}+{\vec{r}}_{P}\right)+{\vec{it}}_{CG}+{\vec{t}}_{P}+{\vec{et}}_{P}.$$

Equation (2) shows how to calculate the vector ${\vec{t}}_{J}$, which represents the torque supported by a joint. In this equation, ${\vec{l}}_{CG}$ is the distance vector between the joint under study and the center of gravity of the isolated segment; ${\vec{l}}_{P}$ is the distance vector between the joint under study and the previous joint along the length of the segment;$\;{\vec{it}}_{CG}$ is the inertial torque, which depends on the angular acceleration, the angular speed, and the inertial tensors; ${\vec{t}}_{P}$ is the torque vector in the previous joint, calculated following this same process; ${\vec{et}}_{P}$ is the external torque applied on the previous joint, which can be entered in the form described in Figure 3; finally, the other terms are those explained for Equation (1).

### 2.5. Risk Calculation Process

Once the human model’s kinematics and kinetics of movement are known, the Forces method can be understood as a score that assesses the risk of suffering musculoskeletal disorders based on this information in the context of the repetitive work cycle. Regarding the concept of the risk of suffering a musculoskeletal disorder, ISO 11228-3 defines certain risk factors that make practical sense. The factors are (1) repetitiveness; (2) strength; (3) posture and movement; (4) duration of work; (5) insufficient recovery time, and (6) additional factors (i.e., vibration, adverse environmental conditions, placement accuracy, and others). Thus, a task repeated many times throughout a day, even if performed with reduced force and accomplished with load postures for a long time without sufficient recovery time and with adverse conditions (e.g., outdoors), presents a high risk of suffering a musculoskeletal disorder.

Based on these factors, Forces calculates the risk for each joint and posture according to the kinematics and kinetics (Factors 2 and 3 of the standard mentioned above) and subsequently obtains a value for each joint, which summarizes the risk during the entire work cycle and includes risks derived from what we call general factors (Factors 1 and 4–6). To conduct this estimation in each joint, Forces applies the following process: (A) First, the biomechanical risk per posture (*RiskPerPosture*) is calculated, which can be understood as the risk value for each posture considering the kinematics and kinetics. The *RiskPerPosture* is a percentage value for the maximum achievable values, established from the experimentation described later in Section 2.6. (B) Subsequently, the total risk per minute (*RiskPerMinute*) is calculated, which is the weighted sum of the risk for all postures. Although the calculation process is linear, and the final result of *RiskPerMinute* is of interest, the *RiskPerPosture* also provides relevant information so that the evaluator can delve into the reasons for the results. 

However, before describing the calculation process, the user must enter specific additional data into the application, the explanation of which helps to understand the calculations. For data entry, a form similar to the one in Figure 3 is used, which includes the following fields:

- Initial and final frame: This is the range of considered frames to calculate the Forces method. If not specified, the entire range of captured frames is considered.
- Cycle time: This is the time granted in seconds by the production area to accomplish the work cycle. If not specified, the time of the initial and final frame range is taken.
- Nonrecovery time per workday: This is the time in hours without rest (whole number). It is considered 1 h if the worker rests at least 10 min. The final hour of the day is always considered recovered.
- Micro-pauses: This is the risk reduction factor that depends on the number of seconds of rest for each manufacturing cycle. According to Rojas and Ledesma [61], it can be considered 1.0 for a cycle without rest, 0.9 for 1 s every three cycles, 0.8 for 1 s every two cycles, and 0.7 for 1 s each cycle.
- Repetitive task time per workday: The time in hours of the workday (decimal number) with physical activity must be entered for the entire workday of the productive area under study. It can be reduced if a period has nonphysical activity during a certain period of the day. In this manner, it is taken by convention that the risk value resulting from a workstation indicates that the worker remains in the workstation throughout the workday. The implications of this convention are extended in the discussion section (see use case).
- Additional factors: This is the percentage of cycle time with additional factors. The value is established at the evaluator’s discretion to consider other factors cited by the ISO 11228-3 standard. The list of additional factors can be consulted in the Supplementary Materials (first sheet in the Excel workbook Forces_Tables.xlsx). For situations in production plants without highlighting factors, it is advisable to set this value at 20% to penalize the results lightly and be on the safe side.
- Worker preparation: This parameter determines the physical condition of the worker who performs the tasks in the evaluated workstation. This parameter affects the maximum achievable values for the risk calculation, which are established according to the methods in Section 2.6. If the value is 0 (sensitive worker), the maximum thresholds (force and torque) are reduced with a coefficient of 0.9 (reduction of 10%). If the value is 1 (average worker), the maximum efforts are not modified. If the value is 2 (trained worker) or 3 (specially trained worker), the maximum efforts are increased with a coefficient of 1.1 or 1.2, respectively. Therefore, this factor can significantly affect the resulting risks. Thus, a value of 1 is recommended unless the prevention service intends to determine whether the risks are still acceptable for a sensitive worker (value 0) or whether the preparation level of the workers is verified or accredited (a value of 2 or 3).

#### 2.5.1. Biomechanical Risk Per Posture

The Forces method uses Equations (1) and (2) to assign a score to the kinematics and kinetics of a joint in each posture and obtain the *RiskPerPosture*. At each frame and in each joint, the risk factors of *AngleScore*, *AngularAccelerationScore*, *ForceScore*, *TorqueScore*, and *GripScore* must be estimated to solve these equations. The minimum value of these factors is 1; thus, as indicated in Equation (3), a *FactorsPerPosture* equal to zero generates a null *RisksPerPosture* percentage. Similarly, as all the factors are multiplied, if a factor takes a value of, for example, 1.5, which implies that the total risk increases by 50%, then each factor affects and contributes proportionally to the resulting total risk:

(3) $$RiskPerPosture\left(\%\right)=\frac{\;FactorsPerPosture\;}{MaximumFactorsPerPosture}\times 100,$$

(4) $$FactorsPerPosture=\left(AngleScore\times AngularAccelerationScore\times ForceScore\cdot TorqueScore\times GripScore\right)-1.$$

**Figure 5:**
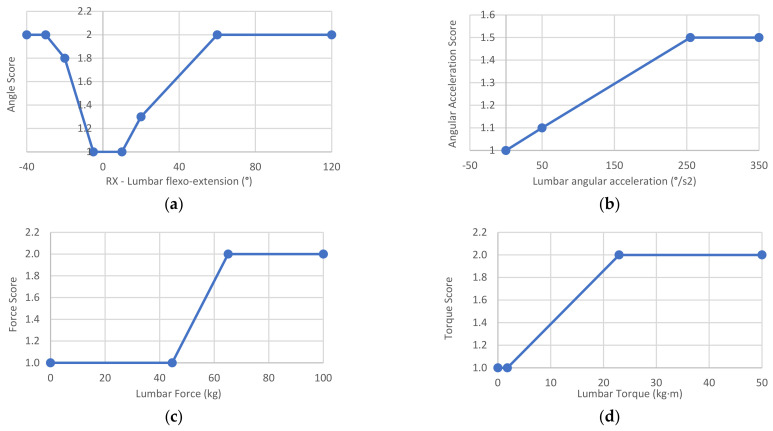
Example of the lumbar joint of the parametric graphs to obtain risk factors introduced in Equation (4). (**a**) *AngleScore*, (**b**) *AngularAccelerationScore*, (**c**) *ForceScore*, and (**d**) *TorqueScore*.

Along this line, the risk factors in Equation (4) are calculated according to parametric graphs, such as those in Figure 5 for the lumbar joint. The complete parametric graphs are presented in the supplementary material; some are inspired from the literature, and some from the experimentation described in Section 2.6. In these parametric graphs, the input value to obtain the risk factors in a particular posture are the values of the joint rotation (to obtain the *AngleScore*), angular acceleration (to obtain the *AngularAccelerationScore*), force module (to obtain the *ForceScore*), torque module (to obtain the *TorqueScore*), and selected grip (to obtain the *GripScore*, which only affects the wrist joint). The following items define each of the factors.

- The *AngleScore* was established to range from 1 to 2 depending on the joint rotations under study; therefore, it can increase the risk by up to 100%. In the case of the lumbar spine, the rotations are flexion-extension (*Rx* of the pelvis, see the human model in Figure 2), rotation (*Ry*), and lateralization (*Rz*). These angles are entered in the corresponding graph in Figure 5a for the lumbar flexion-extension angle. As a result, three scores between 1 and 2 are obtained, one per angle, and the highest score is then introduced in Equation (4). The supplementary material reveals that the shoulder joint is different from the rest due to its wide range of motion. In this case, a double-entry table is used, with the angles of elevation and anteroposterior rotation. The *AngleScore* graphs are inspired by the tables of the main postural loading methods: ISO 11226, REBA [10,11], RULA [12], and OWAS [13]. However, unlike these methods, Forces interpolates between the different points and does not score the angle in steps.
- The *AngularAccelerationScore* was established to range from 1 to 1.5 depending on the angular acceleration of the joint under study; therefore, it can increase the risk by 50%. When the body segment undergoes acceleration or deceleration, the musculoskeletal structures of the involved joint recruit the muscles, tendons, and ligaments necessary to achieve that acceleration and deceleration, which increases the risk on the joint. Therefore, the musculoskeletal requirements on a joint are increased not through speed but change. Thus, each joint has an *AngularAccelerationScore* graph, such as the one in Figure 5b for the lumbar spine, which comes from the experimentation described in Section 2.6. In this graph, the module of the relative angular acceleration is introduced to obtain the score entered in Equation (4).
- The *ForceScore* was established to range from 1 to 2 depending on the module of the force supported by the joint under study; therefore, it can increase the total risk by up to 100%. The graphs defined for each joint must be used to obtain this parameter, which comes from the experimentation described in Section 2.6. Figure 5c displays the example for the lumbar spine for a male model of P50. An internal force in the lumbar spine greater than 65.1 kg (maximum value calculated in the experiment in Section 2.6) corresponds to a *ForceScore* value of 2, and a value less than 44.6 kg (force caused by the body’s weight standing) corresponds to a *ForceScore* value of 1 (minimum value). Intermediate force values are calculated by interpolation, and the resulting value is entered in Equation (4). In this manner, unlike the previous parameters, the *ForceScore* depends on the anthropometric characteristics of the human model. Therefore, the maximum thresholds have a version for the male or female model and P5, P50, and P95 percentiles. In addition, as mentioned above, the *worker preparation* factor also influences the thresholds. All this can be observed in the supplementary material, where a drop-down menu allows the percentile and *worker preparation* level to be selected to view their influence on the graphs. In this manner, in practice, the thresholds for a worker are calculated automatically by interpolation using the height and values included in the supplementary material tables.
- The *TorqueScore* was established to range from 1 to 2.5 depending on the module of the torque supported by the joint; therefore, it can increase the total risk by up to 150%. The interpretation, use, and calculation of the *TorqueScore* factor coincide with the explanation of the *ForceScore* factor. Figure 5d indicates how to estimate the *TorqueScore* for the lumbar joint example.
- The *GripScore* only affects the wrist joint and ranges from 1 to 2; therefore, it can increase the total risk by up to 100%. As described, the grasp type for each action is entered by a drop-down menu using the form in Figure 3. Then, Table 3 is used to obtain the *GripScore*, which is inspired by ISO 11228-3 (OCRA) [8].

After introducing the risk factors, the *MaximumFactorsPerPosture* in Equation (3) can be established. This value is a constant that corresponds to the *FactorsPerPosture* achieved when all factors reach their maximum score. Table 4 lists the *MaximumFactorsPerPosture* for each joint. From this information, it can be deduced that the *FactorsPerPosture* can be between 0 and 14, except for the wrist joint, which can be between 0 and 23.

**Figure 6:**
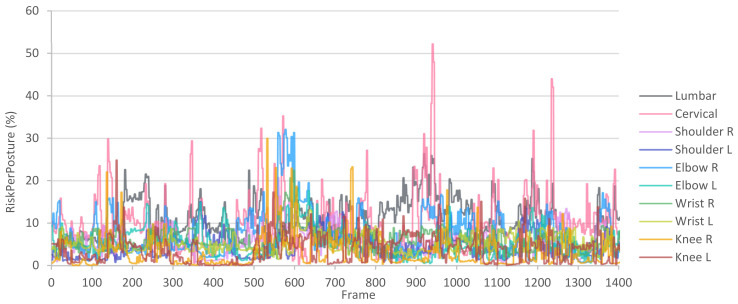
Example of the *RiskPerPosture* in a set of frames. R: Right side. L: Left side.

In this manner, after calculating the *RiskPerPosture* for each posture and joint, a graph like the one in Figure 6 can be plotted. This graph represents the risk of the joints throughout the capture and makes it possible to identify those frames in which the risks are high.

#### 2.5.2. Total Risk per Minute

**Figure 7:**
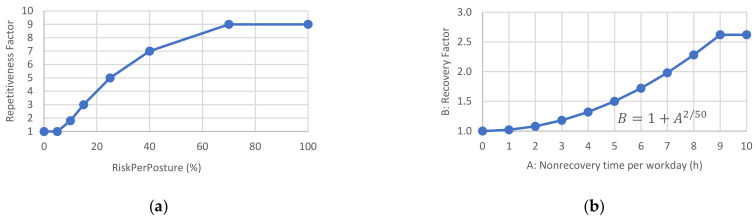

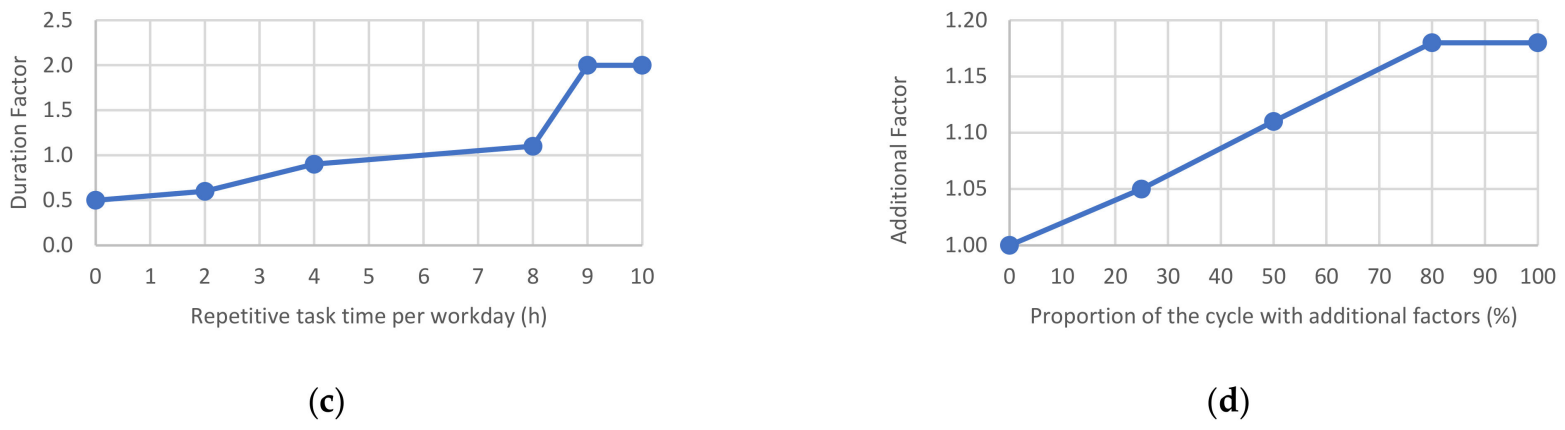
General factor parametric graphs: (**a**) *RepetitivenessFactor*, (**b**) *RecoveryFactor*, (**c**) *DurationFactor*, and (**d**) *AdditionalFactor*.

Afterward, by applying Equation (5), we can calculate the *RiskPerMinute*, representing the final value of the risk for each joint throughout the work cycle. In this equation, the *FactorsPerPosture* for each posture calculated from the previous stage, are summed. Nevertheless, this is a weighted summation; thus, each value of *FactorsPerPosture* is multiplied by the *Repetitiveness Factor*, in Figure 7a. This factor was established to penalize those frames with high risk and prevent the most critical postures from being excessively damped when added to low-risk postures. This factor considers the risk involved in repetitive actions in production cycles throughout the workday:

(5) $$RiskPerMinute\left(\%\right)=\frac{\sum \left(FactorPerPosture\times RepetitivenessFactor\right)\times \frac{60}{CycleTime\;\left(seg\right)}\;}{MaximumFactorPerMinute}\times 100\times GeneralFactors.$$

In Equation (5), the weighted summation described above is multiplied by 60 (seconds per minute) and divided by the cycle time, obtaining the risk weighted per minute, as proposed by ISO 11228-3 (OCRA) [8]. Therefore, for the same number of tasks performed, if the cycle time is reduced, the risk increases and vice versa. Additionally, the *MaximumFactorPerMinute* term in Equation (5) represents the maximum factor that can be achieved in 1 min. This factor is a constant calculated in Equation (6), which multiplies the *MaximumFactorPerPosture* described in the previous section by the 3600 postures processed per minute (60 postures per second):

(6) MaximumFactorPerMinute=MaximumFactorPerPosture×3600.

The *GeneralFactors* term is introduced to complete Equation (5). As mentioned, certain risk factors exist other than those derived from kinematics and kinetics, which can cause or aggravate work-related musculoskeletal disorders. The Forces method considers these factors under the *GeneralFactors* concept, which is calculated using Equation (7):

(7) $$GeneralFactors=\left(RecoveryFactor\times MicroPausesFactor\right)\times DurationFactor\times AdditionalFactor.$$

In Equation (7), the *RecoveryFactor* depends on the nonrecovery time per workday (whole number of hours) entered by the user in the form (see the beginning of Section 2.5). The factor can range from 1 to 2.6 and is calculated according to Figure 7b. The *RecoveryFactor* can be reduced by the *MicroPausesFactor*, which is between 0.7 and 1 and, as indicated above, is entered by the user.

The *DurationFactor* is calculated according to the graph in Figure 7c as a function of the amount of repetitive task time per workday (decimal number of hours), which the user enters. This factor can vary from 0.5 to 2, although it typically does not exceed a value of 1.1, corresponding to 8 h of physical work.

Additionally, the *AdditionalFactor* is calculated in Figure 7d. This factor depends on the proportion of the cycle with additional factors described above, entered at the user’s discretion. This factor can vary between 1 (no additional factors) and 1.18, which would be the most unfavorable situation.

#### 2.5.3. Final Risk Assessment

According to the above, the *RiskPerMinute* represents the total risk for each joint throughout the work cycle. Table 5 includes the *RiskLevel*, which can range from 0 to 5 and is calculated by interpolating the *RiskPerMinute* value reached for each joint according to the intervals in the table and the resulting valuation and interpretation. In order to improve the interpretation process, a color is assigned to each *RiskLevel*. These scoring, color, and assessment levels are inspired by the ISO 11228-3 (OCRA) standard [8].

### 2.6. Experimentation to Obtain the Maximum Risk Database

An experiment was conducted with seven healthy volunteers (four males and three females of 36.7 ± 15.3 years old) to develop the described *ForceScore*, *TorqueScore*, and *AngularAccelerationScore* parametric graphs included in the supplementary material. The Bioethics Committee of Aragón, Spain (N° 12/2018) approved the study, and informed consent was obtained from all participants.

In this experimentation, three types of exercises were conducted. The first type was to capture the maximum stresses (force and torque) and the maximum angular accelerations of each joint. The second type was to record the stresses at rest in a standing position. Finally, the third was to measure the minimum angular accelerations.

The estimation of stresses and accelerations was accomplished using the MH system and described kinematic and kinetic calculations. Additionally, regarding the protocol, IMU sensors were placed on the participants as described by Marín et al. [35] and presented in Figure 1. Then, a researcher stood in front of the participant to indicate the movements to perform in each exercise.

For the first type of exercise, each participant described wide arcs with each joint under study: lumbar, cervical, shoulders, elbows, wrists, and knees. The movements were executed to reach angular maximums in all rotation axes. The supplementary material includes a set of photograms showing representative postures performed by the volunteers during the experimentation. The velocity was previously agreed upon by the researchers and was planned to represent the maximum speed reached in work actions without achieving speeds of other activities, such as sports.

Additionally, to achieve more realistic movements during this exercise, the volunteers carried dumbbells with a weight of 2 kg in each hand. Nevertheless, before processing these captures, it was assumed that the participant was carrying a load of 8 kg in each hand instead of 2 kg, resulting in a total load of 16 kg, which is considered the maximum load to be manipulated [9]. For this purpose, the form in Figure 3 was used, assuming a force vector of (0, −8, 0) in each hand throughout the capture. Therefore, the results can be considered maximum stresses and accelerations for ergonomic evaluation. Furthermore, they represent the most unfavorable situations by manipulating weighted objects.

Regarding the amount of information collected in this first experiment, the seven participants performed movements for about 3 min, during which they moved all joints. Each 3-min capture was repeated three times for each participant. Therefore, a total of 7 × 3 = 21 captures were performed. Likewise, as described, the MH application allows the human model to be subsequently modified to a male or female model of percentile P05, P50, or P95 (i.e., six combinations of models). The result is as if the captured movement had been performed by a man or woman with the anthropometry of these percentiles. This process of changing the human model was accomplished with these 21 captures. Consequently, a total of 21 × 6 = 126 captures were processed for the analysis of the maximum stresses and accelerations.

Subsequently, the second type of exercise was performed. In this exercise, a resting posture was captured in the standing position for a few seconds without any weight on the hands to obtain the minimum values of the stresses. The resting position already produces stresses on the joints; for example, the lumbar spine must support the weight of the trunk, head, and upper extremities.

Afterward, the third type of exercise, which consisted of the same exercises as in the first type, was performed but with slow speeds without a weight on the hands. The researchers agreed on the velocity to perform the movements during this capture to avoid increasing the risks of suffering musculoskeletal disorders caused by inertia from moving the body segments. In the second and third types of exercise, three captures were taken with one participant and transformed according to various human models.

## 3. Results

This section presents the results of the experiment described in Section 2.6, allowing the composition of parametric graphs relative to *ForceScore*, *TorqueScore*, and *AngularAccelerationScore*. The complete parametric graphs are in the supplementary material.

Table 6 includes the results of the variables presented in the following bullet points, which are computed for each capture of male (M), female (F), and percentile (P50, P05, and P95). In this table, for each variable and joint, the average value and its standard deviation in brackets are presented. Note that the *AngularSpeed* variables do not change between male, female, or percentiles because they do not depend on the dimensions of the human model, only on the relative angles measured by the sensors.

- *Force Max* and *Torque Max:* module of the force and torque corresponding to the 99th percentile of the set of values resulting from capturing the first type of exercise;
- *Force Min* and *Torque Min:* average of the module of the force and torque resulting from a static capture in the standing position (i.e., the second type of exercise);
- *Angular Speed Max:* module of the angular velocity corresponding to the 99th percentile of the set of values resulting from capturing the first type of exercise;
- *Angular Speed Min:* average angular speed during slow gesture capture (i.e., the third type of exercise).

The results of Table 6 are transformed into parametric graphs. For this purpose, values obtained on the right and left sides are averaged, and the coefficients to obtain the P05 and P95 (CoefP05 and CoefP95) from the P50 are calculated. These coefficients, are the ratio between the values obtained for the P50 or P50 and the P50 included in Table 6. Note that the minimum force and torque values of Table 6, which correspond to the static experimentation (second type of exercise), are only calculated for the 50th percentile. For their conversion to other percentiles, the same transformation coefficients (CoefP05 and CoefP95) resulting from the dynamic experimentation (first type of exercise) are used.

Thus, Table 7 and Table 8 list the data that define the *ForceScore* and *TorqueScore* parametric graphs. As described, the maximum values at each joint define the threshold above which the risk is maximum. The minimum values correspond to the stresses in the joints in a resting standing position with no load on the hands.

Finally, Table 9 presents the information that defines the *AngularAccelerationScore* parametric graphs. The minimum values refer to an acceleration below, which there is considered to be no risk, and the maximum values refer to an acceleration above which the risk factor is maximum. It was considered appropriate to start from the angular speed to calculate the acceleration because it is more reasonable to assess. In this manner, the angular acceleration was estimated considering that the joint at these angular speeds stops in 0.2 s (12 frames at 60 fps, i.e., *Angular Acceleration* (°/s^2^) = *Angular Speed* (°/s)/0.2 (s)).

## 4. Discussion

This paper presents the rationale and justification for the Forces ergonomic method, which is designed to assess the risk of musculoskeletal disorders in a worker performing repetitive and load handling tasks. It is a direct-measurement ergonomic assessment method designed to be applied in situ with a portable motion capture system, in our case, the MH system. This method provides an automated risk estimation. The risk assessment is based on estimating joint stresses resulting from the postures, velocities, and accelerations of the captured movement and the external forces that the worker exerts during the work cycle. This stress estimation and the general factors related to the organization and context make it possible to estimate the risk in different anatomical areas (lumbar, cervical, shoulders, elbows, wrists, and knees).

The concept of risk in the Forces method is understood as a percentage with respect to the maximum thresholds established by experimentation (i.e., how much the joint is exposed in relation to the maximum admissible amount for each posture (*RiskPerPosture*) and the overall work cycle (*RiskPerMinute*)). This scoring system provides a predictive method of suffering musculoskeletal disorders, which aids in prioritizing ergonomic interventions aimed at the workers’ behavioral or postural education and the workstation design or redesign.

Concerning the experimentation presented to calculate the maximum stresses, the multidisciplinary team defined the movements jointly. Thus, both the execution of the gestures and the inclusion of a load of 8 kg in each hand generated realistic results to establish the maximum thresholds. The values obtained on both sides, right and left, are practically identical, and the standard deviations are remarkably low, which indicates that the collected data are homogeneous and stable in the studied subject sample. These values represent thresholds that should not be exceeded during the execution of repetitive work. Thus, although future experiments can be conducted to provide more information, for the moment, these values represent a reasonable and realistic approximation.

Concerning the structural validity of the method we intend to achieve [42,45,46], as described above, the Forces method must be effective and efficient. Given Section 2, where the fundamentals of the method are set out, and the results in Section 3, we can affirm that, in terms of effectiveness, Forces fulfills its stated purpose of calculating risks through a logical, justified, structured procedure without redundancies or errors. However, to complete its structural validity, we must discuss whether the method is efficient (i.e., useful and applicable in its context).

To address this last point, in Section 4.1, we discuss the value of the method compared with the existing methods. Then, in Section 4.2, a use case is presented, explaining the application of Forces to a workstation and a set of workstations. Afterward, Section 4.3 discusses the contribution of forces to the concept of Ergonomics 4.0 or smart ergonomics [22,23,24,25]. Finally, Section 4.4 discusses the limitations and future actions.

### 4.1. Value Compared with the Existing Methods

Based on the outlined fundamentals, Forces is an ergonomic evaluation method that advances the current observational methods. Its initial design is focused on the whole information and possibilities from a motion capture system, and although observational methods inspire it, it is not an adaptation of an existing method [8,9,10,11,12,13,14,15]. Therefore, it provides a method that frees the evaluator from tedious tasks required by traditional observational methods and uses an automated joint risk scoring system that is not influenced by the subjectivity of the assessor.

Concerning the risk due to postural load, Forces provides a risk similar to the aforementioned observational methods, but in this case, considering the stresses caused by movement. Additionally, it performs the calculation at 60 postures per second, which provides added value over the traditional postural load assessment methods, which are limited to instances chosen by the assessors, depending on which instance they consider to present a higher risk according to their previous experience and knowledge.

Regarding the risk of load handling, Forces calculates the internal stresses that the joints must withstand to perform the required actions (forces and torques). These stresses consider the inertias caused by the body movement, the inertias derived from the mass of the handled object, and the acceleration or deceleration that the worker performs during handling. In addition, they are estimated in the intermediate postures between picking up and putting down the load, which can also be harmful to the worker and is essential to consider when assessing the risk of handling loads. Consequently, it adds significant value to applying such methods as the NIOSH equation (ISO 11228-1) [9].

In another vein, the method also accounts for linear forces in any direction and torques exerted with the hands in any axis of rotation [57,58,59,60]. Thus, this method provides analysis possibilities beyond the usual vertical forces exerted by load manipulation and allows considering a wide range of situations in practice.

Finally, the method allows running simulations. In other words, it permits the modification of parameters such as external forces, torques, or even the percentile of the human model, to observe the effect on the resulting risk estimation. This approach is particularly relevant to anticipate specific improvement actions applied to the workstations under study [62,63].

### 4.2. Manufacturing Industry Use Case

Regarding the applicability and usefulness of the method, in this section, we present a use case related to the consumer goods manufacturing industry, where workstations with repetitive tasks and load handling are typical. For this purpose, let us consider that we intend to assess five workstations (coded from P001 to P005) located in a production line that manufactures 120 washing machines per hour. These workstations belong to a section of this line where five workers rotate throughout the workday. The working hours are from 6:30 a.m. to 2:30 p.m., with 20-min breaks at 10:00 a.m. and 12:00 p.m. The environmental conditions are favorable; it is a temperature-controlled warehouse without excessive noise, vibration, or excessive work pressure.

In this context, it is possible to define specific data for the Forces application relative to the production plant and applicable to the five stations. We consider 30 s of cycle time (120/60 = 2 washers every minute), 5 h without recovery, 0.8 micro-pauses (1 s of rest every two cycles), 7.33 h (440 min) of repetitive activity, and 20% for additional factors (established value for productive plants without adverse conditions).

In this scenario, we use the motion capture system to record the workers’ movements at the workstations. To do this, we select one of the five workers, preferably the most experienced, with a size and weight as close as possible to P50, whom we call “Jaime”. To avoid interrupting production, another worker should replace Jaime because he will be occupied making the recordings. In this manner, Jaime replaces each of the five workers to capture the work cycle, and then the regular operators continue the work.

During the measurement protocol, it is recommended that two assessors operate the capture system: one to control the computer and one to manage the portable camera. The handheld camera records the tasks Jaime performs with his hands, which is necessary to apply the method together with the recorded movement. With this, the sensor placement time is about 6 min, and the explanation and adaptation of the worker to the technology is about 8–10 min. The capture time corresponds to the cycle time, adding the time to move between stations and the possible wait for some of them to evaluate the production situation, which may depend on the manufactured product model and the filling of the containers, among other factors. In total, we can assume between 60–90 min for recording the five stations.

Once motion capture has been performed and the production plant information has been entered into the system, we use the form in Figure 3 to enter external forces exerted by the worker in each workstation. Let us take the example of the P003 workstation, which we assume requires the worker to (1) take the washing machine drum (known weight of 3.5 kg) from a pallet and position it on the washing machine structure located on the line. (2) Then, the worker places two screws on the front with an automatic screwdriver that is suspended. (3) Next, the worker takes two other pieces, located on each side, and inserts them into the product. These pieces have no appreciable weight and require no representative force for insertion. (4) Finally, the worker presses a foot pedal to register the end of the cycle.

In this manner, in the P003 form, we include an action for manipulating the load and two actions for the screws. In the load manipulation, we fill in the first two fields of the form by observing the video frames in which the load is picked up and released, for example, from frames 512 to 653. With this, we introduce a force vector of (0, −3.5, 0) and a grasp type of “4—Reasonably appropriate hook” because he picks up the drum with his fingers and, although the piece has good proportions, it does not have a grip designed to be manipulated. For the screwing actions, we search the frames in the video following the same operation as in the load manipulation. For the following fields, in this case, it is not necessary to enter the tool weight because it is suspended. However, we must enter the torque of this tool according to its technical specifications; we assume it is 0.2 kg∙m. Further, as it is screwed forward, we include a torque vector of (0, 0, −0.2), which the operator perceives due to the use of the tool, and a grasp type of “1—Appropriate wrap.”

**Figure 8:**
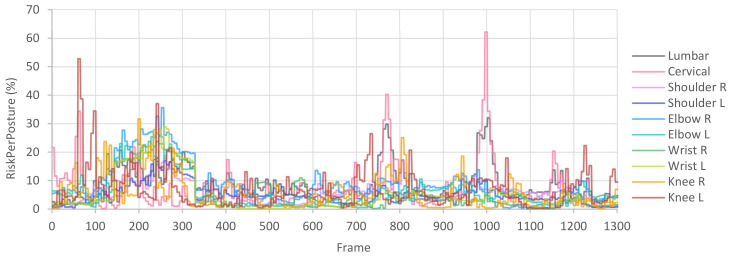
Results of the P003 workplace example: *RiskPerPosture* graph.

Therefore, for the P003 workstation, we obtain the *RiskPerMinute* for each joint included in Table 10. We observe that the highest risks are in the left elbow, implying a “conditional” rating (Table 5). If it had exceeded 40%, it would be an “unacceptable” rating and would require corrective actions. The subsequent highest risks are in the lumbar and cervical joints, but they have an “acceptable” rating. Likewise, we can consult the *RiskPerPosture* graph in Figure 8, where we observe the evolution of the risk of all joints evaluated throughout the manufacturing cycle. In this case, in the frame interval 0 to 300, where load handling occurs, the risk to several joints increases. Subsequently, during interval 300 to 700, where screw actions occur, the risks decrease, demonstrating that these actions do not involve an appreciable load on the joints. Then, in the intervals 700 to 900 and 900 to 1100, where the right and left parts are picked up, the lumbar and cervical risks increase due to back flexion. Finally, from frame 1100 onwards, where the pedal is pressed, the risks decrease again.

Next, to be more realistic, we assume that the drums are stacked seven high on the pallet at workstation P003, implying that the risk varies if the worker picks up the drum in the lowest or highest position. In these cases, it is recommended to capture the most representative situations to obtain an overall workstation assessment. Therefore, we conducted three captures, one handling the drum from an intermediate height (version P003_Medium, results described in Table 10), another handling the drum from the upper area (P003_Top), and another from the lower area (P003_Low).

As illustrated in Table 11, and as expected, the risks are slightly different for each situation. When the drum starts in the lower zone, the risks in the lumbar zone become maximum (yellow) due to flexion. To obtain an average *RiskPerMinute* value for the workstation, we combined the risks in these situations. To do this, we propose a weighted sum of the risks, assigning a weight (*W*) to each captured situation. As there are seven heights, we assume that the three intermediate heights are similar to the P003_Medium situation (3 out of 7, 42.8%), the two upper heights are similar to the P003_Top situation (2 out of 7, 28.6%), and the two lower heights are similar to the P003_Low situation (2 out of 7, 28.6%). The results are displayed in Table 11, representing the combined risk of the assumed situations, which we call P003_COMBI.

Proceeding in the five workstations in the same manner as in workstation P003, we obtained the risk map [26] of Table 12, where we can view the resulting risks in each joint for each workstation. This risk map is beneficial for detecting workstations with more significant ergonomic risks that require more attention. In this regard, the risk map lists the results for each workstation, assuming that the worker is assigned to only this workstation during the entire workday. This agreement is useful for verifying whether the risk for each workstation is acceptable.

Nevertheless, if we intend to assess the benefits of a specific job rotation during the workday, we can proceed similarly to the described combination process. We can create a weighted sum of the risks of the workstations, assigning the corresponding time of the workday to each of them (Table 12). In the example, if the workers spend the same time at all workstations (440 min/5 workstations = 88 min per workstation), we assign 20% to each of them. This calculation is inspired by the multitask ISO 11228-3 (OCRA) standard [8] and does not consider the order of the tasks. Although the order could affect the recovery of some musculoskeletal regions, the purpose of this simulation is to validate an acceptable job rotation within a workday. Additionally, an individual workstation may have unacceptable risk if performed throughout the day (as observed in workstation P004). However, the resulting risk may be acceptable when combined in a job rotation, which is the intended effect of mitigating risks through job rotation throughout the workday.

The weighted sum of the *RiskPerMinute* values can be calculated without introducing the *RecoveryFactor*, *MicroPausesFactor*, and *DurationFactor* in the equations and then multiplying the result by these factors to be more consistent and follow the basis of the ISO 11228-3 (OCRA) standard [8] because they are multitasking factors. However, we propose to do it as described because multiplying before or after does not affect the result, and it is more complex to manage two risk maps, with and without considering multitask factors.

According to the above, the Forces method is valuable and applicable in its context and has practical value. Therefore, it is an efficient method with several practical advantages. It is objective, and the processing is automated. In addition, it is designed considering the technology possibilities and leverages all collected information. Moreover, it does not require excessive time in measurement, processing, or interpretation, and (5) does not demand high knowledge for its use. Further, (6) it provides an overview of the evaluated workstations (*RiskPerMinute* values included in the risk map) and allows drilling into details to detect the causes (*RiskPerPosture* graph). Additionally, it considers external factors, such as working hours or the work environment, and it permits running simulations by varying parameters and reprocessing to observe the effects. Finally, it allows different versions of a workstation to be combined and facilitates the study of job rotations.

### 4.3. Smart or 4.0 Ergonomics

**Figure 9:**
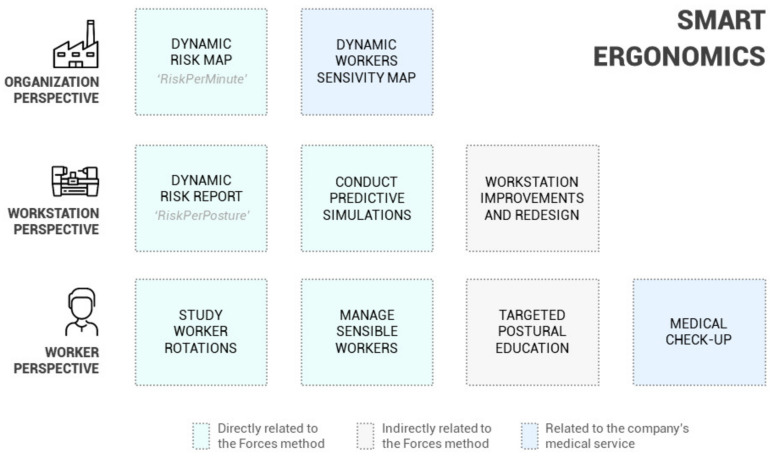
Relevant ergonomic actions for intelligent ergonomics and its connection with Forces.

Given the discussion in the previous sections, the Forces method provides a structurally valid methodology to promote and enhance intelligent or 4.0 ergonomics [22,23,24,25]. However, to further explore how and to what extent Forces is integrated into smart logic and Industry 4.0 [17], the schematic in Figure 9 is presented and described in this section.

Figure 9 lists specific ergonomic actions relevant to achieving intelligent ergonomics and their connection with the Forces method at a conceptual level. These actions are organized from three perspectives: organizational actions conducted from a management approach, workstation actions focused on the design or study of the handled physical elements, and worker actions aimed at improving the health and well-being of workers. The activities are presented in closed boxes; however, they are interconnected.

Likewise, in this scheme, actions are categorized in three colors. The first category comprises those directly related to Forces, as they are direct functionalities of the method. The second includes those indirectly related to Forces, as they can be accomplished based on the results, but their execution does not depend on the method. Finally, the last category consists of those related to the medical service and follow-up of workers’ health, which have an essential role in risk prevention [64].

The organizational perspective of Figure 9 includes working with “dynamic risk maps” and “dynamic worker’s sensitivity maps.” As explained in Section 4.2, the risk maps correspond to tables presenting an overview of the workstation and the associated risks for each joint. These maps are generated directly after the application of Forces. Moreover, the worker sensitivity maps identify those workers with musculoskeletal injuries or problems and the affected anatomical areas. These maps come from periodic medical check-ups or injuries reported by workers [65]. From the organizational perspective, being able to contrast and relate both maps can be considered a basis of intelligent ergonomics [26]. If it becomes possible to associate the workstation features with the workers’ particularities, it would be possible to personalize the task assignment from an ergonomic approach and significantly reduce risks and injuries.

Both maps include the “dynamic” concept (i.e., they are not static in time and must be continuously updated [26]). In worker sensitivity maps, their dynamism is evident, as health status can change continuously. The workstation risk maps can change, either because workstation conditions change and require ergonomic reassessment or because specific organizational parameters change and affect the Forces calculation.

In this regard, it would be advisable that the workers’ future sensitivity maps include reported injuries and also an evaluation of the worker’s capacity (e.g., with a periodic functional motion capture test [66,67]). Additionally, if this measurement of capacity or sensitivity level had the same format as the risk map (i.e., divided by joints and a percentage value), it would allow contrasting both maps in a more transparent, orderly, and straightforward manner, generating a more efficient decision-making process.

Shifting the perspective, the workstation actions of Figure 9 refer to integrating the productive requirements and also the ergonomic and preventive needs into the design of workstations [65]. Detecting ergonomically unacceptable workstations through the risk maps leads to questioning the causes, which requires analyzing the results more deeply, for which Forces presents the *RiskPerPosture* graph. This graph allows detecting which postures cause the most significant problems and facilitates identifying specific problems that must be redesigned, which is one of the critical actions in implementing intelligent ergonomics [68,69]. For this purpose, as mentioned, Forces allows simulations (e.g., change human model percentile, vary the forces introduced, etc.), simplifying testing and validating different possibilities and reaffirming that Forces is not intended for one-time use but continuous use as a working tool within an intelligent ergonomic action framework.

Finally, concerning the worker’s perspective in Figure 9, the ergonomic actions should also be aimed at managing and training workers. These actions should be complemented and coordinated with the company’s health surveillance service without omitting periodic medical check-ups. In this frame, two fundamental actions exist regarding the actions to be accomplished for workers: job-rotation management, where we include the appropriate location of sensitive workers, and postural education.

Job rotation reveals how Forces allows organizing balance rotations that do not overload specific joints, facilitating the relocation of workers with specific injuries to assign workstations with low risk in the affected anatomical areas. In this regard, rotations may have specific initial barriers, such as short-term cost, the negative psychosocial response of workers, or reticence to leave the usual workstation [70]. However, rotation is associated with reducing the severity and incidence of musculoskeletal disorders and an improvement in long-term productivity. Furthermore, rotation reduces exposure to postural demands from high-risk workstations, reducing psychological stress, mitigating the fatigue of specific muscle groups due to repetitiveness, and increasing production flexibility due to less dependence on expert workers [70,71,72,73].

Regarding postural education, the value that Forces provides is the detection of specific problems. The method facilitates focusing the training on the specific issues, thus avoiding generic training sessions that do not represent the real needs. Both education in ergonomics and prevention make it possible to understand the risks underlying the performance of specific tasks. Proper education should, among other things, raise awareness of preventive measures, provide a strategy to identify which factors can trigger injuries or postural disorders, and transmit certain basic principles of action [65]. In this aspect, motion capture technology, virtual reality, postural simulation, and biofeedback-based training can have an important role [74,75].

From the exposure, Forces constitutes a working support tool for various professionals: risk prevention technicians, occupational physicians, therapists, quality technicians, human resource managers, and more generally, professionals involved in disciplines related to ergonomics or biomechanics. It provides a methodology that allows industrial companies to apply an intelligent ergonomic action through anticipatory actions and a predictive model. This method aids in the preservation of the worker’s health and prevents possible disorders that cause temporary or chronic disabling injuries, which cause considerable business and social costs. In this manner, given the connection between product quality and the quality of the working conditions [1], Forces benefits both worker health and the productivity and competitiveness of companies.

### 4.4. Limitations and Future Work

First, a limitation of this study is related to the subject sample, which is seven volunteers. However, several captures were made per subject, and each was multiplied due to the change in the human model for gender and percentiles, resulting in 126 processed captures, which is a considerable number. Additionally, the deviations obtained in the experimental results are minimal. Thus, although future studies may be conducted with a larger subject sample, the results are sufficient and constitute an adequate starting point for applying the proposed method.

Regarding the IMU technology used, it should be noted that the Fitbody calibration process allows the magnetometers to be disabled for short-term captures. Nevertheless, due to the drift error that increases with time in the absence of magnetic information, this process has the limitation of losing precision in longer captures. In this regard, the most straightforward approach for resolving the drift errors is to limit the capture time, which is appropriate when only a short period is required to execute the movements being investigated [76]. Nevertheless, it would be helpful to conduct future studies to evaluate the loss of precision over time and the method’s sensitivity to joint angle drift. Additionally, to prolong the capture duration, one approach is to use the exploitation of kinematic constraints (such as boundary conditions in the degrees of freedom or the range of motion of the joints), since they limit the drift artifact [77,78]; another possible approach is to use zero-velocity updates [79] or dead reckoning [80] methods, which reset integration and acceleration errors when detecting zero-velocity periods during the steps.

Furthermore, with the perspective of going beyond the structural validity presented here, other evaluation actions can be accomplished. To this end, we understand that it does not make much sense to compare this method with the observational methods because it is directly inspired by them. If these observational methods were applied throughout all postures, which would be an extremely laborious task, the data would have a similar tendency. Perhaps to consolidate Forces more effectively, it would be better to conduct epidemiological studies in actual production environments. These studies would contrast the predictions of musculoskeletal injuries made by the Forces method with the opinion of workers and managers regarding workstation demands and with information from medical services regarding the workstations causing the most injuries or discomfort. This contrast would allow adjusting the values of the method to achieve a better prediction, which can be materialized in new versions of the supplementary material tables.

Finally, concerning its application, the Forces method is not a tool that provides a solution per se to the problems and is not instantaneous but requires an iterative process of capture, analysis, and interpretation; however, its regular and continuous application would bring value to preventing musculoskeletal disorders, especially if complemented and coordinated with health surveillance services. All this would reduce the economic losses caused by musculoskeletal disorders in the working population, which the company and society must bear.

## 5. Conclusions

The presented Forces method is an ergonomic method of direct measurement, which estimates the risk of suffering musculoskeletal disorders in the anatomical areas of the cervical, shoulders, elbows, wrists, and knees in a worker performing repetitive tasks or handling loads. It is applied in situ at the workstation with a portable inertial sensor motion capture system and provides an automated risk estimation. The risk assessment is based on the estimation of joint stresses, the captured movement, the external forces performed by the worker during the work cycle, and certain general factors related to the organization and context. It is a method designed considering the possibilities offered by motion capture technology and its application in industrial environments.

This study concludes that the Forces method is structurally valid in terms of effectiveness and efficiency. The method is effective because, according to its fundamentals, it fulfills its stated purpose of calculating risks by employing a logical, justified, structured procedure without redundancies or errors. It is efficient because it adds value concerning the existing methods, has practical value, is useful and applicable in its context, and contributes to smart ergonomics. With all this, this method constitutes a working support tool for today’s industry, reducing musculoskeletal disorders derived from repetitive tasks and the social, economic, and productivity losses that such disorders entail.

## Figures and Tables

**Table 1:** Height and weight percentiles of the population according to Huston [54].

	Male Height (cm)	Male Weight (kg)	Female Height (cm)	Female Weight (kg)
P05	164.9	66.21	151.8	49.44
P50	175.9	80.50	161.8	59.85
P95	186.9	96.41	172.4	72.43

**Table 2:** Values of %*W*, %*CG*, and *T* for the kinetic calculation according to Huston [54].

	*%W* M	*%W*F	*%CG*M	*%CG*F	*T* M.P05	*T* M.P50	*T* M.P95	*T* F.P05	*T* F.P50	*T* F.P95
Pelvis	12.42	16.72	50.0	50.0	[0.065, 0.105, 0.105]	[0.091, 0.148, 0.148]	[0.122, 0.199, 0.190]	[0.052, 0.083, 0.086]	[0.075, 0.120, 0.123]	[0.106, 0.169, 0.174]
Lumbar	13.60	11.02	50.0	50.0	[0.065, 0.105, 0.105]	[0.091, 0.148, 0.015]	[0.122, 0.199, 0.199]	[0.031, 0.050, 0.051]	[0.045, 0.072, 0.074]	[0.064, 0.102, 0.104]
Thorax	23.19	15.55	49.7	49.7	[0.052, 0.075, 0.075]	[0.073, 0.106, 0.106]	[0.098, 0.142, 0.142]	[0.021, 0.031, 0.031]	[0.030, 0.044, 0.044]	[0.042, 0.062, 0.062]
Head	8.39	9.13	30.0	30.0	[0.027, 0.014, 0.027]	[0.038, 0.019, 0.038]	[0.051, 0.026, 0.051]	[0.017, 0.009, 0.017]	[0.025, 0.013, 0.025]	[0.035, 0.018, 0.035]
Arm	2.77	2.85	38.0	37.9	[0.002, 0.019, 0.019]	[0.003, 0.027, 0.027]	[0.004, 0.036, 0.036]	[0.001, 0.012, 0.012]	[0.002, 0.017, 0.017]	[0.003, 0.024, 0.024]
Forearm	1.73	1.71	49.5	49.6	[0.001, 0.014, 0.014]	[0.001, 0.020, 0.020]	[0.001, 0.027, 0.027]	[0.001, 0.009, 0.009]	[0.001, 0.013, 0.013]	[0.001, 0.018, 0.018]
Hand	0.65	0.69	80.0	80.0	[0.001, 0.001, 0.003]	[0.001, 0.001, 0.004]	[0.001, 0.001, 0.005]	[0.001, 0.001, 0.001]	[0.001, 0.001, 0.002]	[0.001, 0.001, 0.003]
Thigh	10.49	12.57	53.2	53.0	[0.069, 0.017, 0.069]	[0.097, 0.024, 0.097]	[0.130, 0.032, 0.130]	[0.049, 0.013, 0.049]	[0.071, 0.018, 0.071]	[0.100, 0.025, 0.100]
Shin	4.28	4.53	49.8	49.4	[0.006, 0.001, 0.006]	[0.008, 0.001, 0.008]	[0.011, 0.001, 0.011]	[0.003, 0.001, 0.003]	[0.005, 0.001, 0.005]	[0.007, 0.001, 0.007]
Foot	1.28	1.44	50.0	50.0	[0.005, 0.005, 0.001]	[0.007, 0.007, 0.001]	[0.009, 0.009, 0.001]	[0.003, 0.003, 0.001]	[0.004, 0.005, 0.001]	[0.006, 0.007, 0.001]

Notes: %*W*: percentage of weight by body parts, %*CG*: position of the centers of gravity as a percentage of the length of the bone, concerning its origin, *T*: inertial tensors, M: male, F: female.

**Table 3:** *GripScore* definition.

Grasp Type	*GripScore*
0. Not specified	1.0
1. Appropriate wrap	1.3
2. Unprepared wrap	1.8
3. Appropriate hook	1.3
4. Reasonably appropriate hook	1.6
5. Unprepared hook	1.9
6. Pinch	1.3
7. Precision pinch	1.6
8. Appropriate open hand	1.7
9. Unprepared open hand	2.0

**Table 4:** *MaximumFactorPerPosture* for each joint.

	*Angle* *Score*	*Angular* *Acceleration Score*	*Force* *Score*	*Torque* *Score*	*Grip* *Score*	*Maximum* *Factors Per Posture*
Lumbar	2.0	1.5	2.0	2.5	1.0	14
Cervical	2.0	1.5	2.0	2.5	1.0	14
Shoulder	2.0	1.5	2.0	2.5	1.0	14
Elbow	2.0	1.5	2.0	2.5	1.0	14
Wrist	1.6	1.5	2.0	2.5	2.0	23
Knee	2.0	1.5	2.0	2.5	1.0	14

**Table 5:** Scoring and interpretation of the risk reached at a joint throughout the entire work cycle.

*RiskPerMinute* (%)	*RiskLevel*	Valuation	Interpretation
≤10	≤1	No risk	Acceptable
>10 ≤ 15	>1 ≤ 2	Low risk	
>15 ≤ 25	>2 ≤ 3	Medium risk	
>25 ≤ 40	>3 ≤ 4	High risk	Conditional
>40 ≤ 70	>4 ≤ 5	Very high risk	Unacceptable
>70	>5	Severe risk	

**Table 6:** Experimentation results.

Variable	P (%)	Lumbar	Cervical	Shoulder R	Shoulder L	Elbow R	Elbow L	Wrist R	Wrist L	Knee R	Knee L
M. *Force* *Max.* (kg)	P50	65.1 (0.8)	8.8 (0.0)	13.6 (0.1)	13.6 (0.1)	11.6 (0.0)	11.6 (0.0)	10.6 (0.1)	10.6 (0.1)	81.1 (6.7)	80.2 (10.2)
	P05	56.4 (0.3)	7.2 (0.0)	13.0 (0.1)	13.0 (0.1)	11.4 (0.1)	11.4 (0.0)	10.5 (0.1)	10.5 (0.1)	68.0 (5.7)	67.0 (8.6)
	P95	75.3 (1.4)	10.5 (0.0)	14.3 (0.1)	14.3 (0.1)	11.9 (0.0)	11.9 (0.0)	10.7 (0.1)	10.7 (0.1)	95.8 (7.4)	95.0 (11.7)
M. *Force* *Min.* (kg)	P50	44.6 (0.0)	6.8 (0.0)	4.2 (0.0)	4.2 (0.0)	1.9 (0.0)	1.9 (0.0)	0.5 (0.0)	0.5 (0.0)	36.4 (0.3)	37.5 (0.5)
F. *Force* *Max.* (kg)	P50	46.9 (0.2)	7.1 (0.0)	12.7 (0.1)	12.7 (0.1)	11.2 (0.1)	11.2 (0.0)	10.5 (0.1)	10.5 (0.1)	60.8 (5.7)	60.0 (7.9)
	P05	42.0 (0.2)	5.9 (0.0)	12.3 (0.1)	12.3 (0.1)	11.0 (0.1)	11.0 (0.1)	10.4 (0.1)	10.4 (0.1)	51.5 (5.1)	50.8 (6.8)
	P95	53.0 (0.3)	8.6 (0.0)	13.3 (0.1)	13.3 (0.1)	11.5 (0.1)	11.5 (0.0)	10.5 (0.1)	10.5 (0.1)	72.2 (6.5)	71.3 (9.4)
F. *Force* *Min.* (kg)	P50	27.7 (0.0)	5.5 (0.0)	3.1 (0.0)	3.1 (0.0)	1.4 (0.0)	1.4 (0.0)	0.4 (0.0)	0.4 (0.0)	26.8 (0.2)	27.6 (0.4)
M. *Torque* *Max.* (kg∙m)	P50	22.95 (2.79)	0.90 (0.07)	5.86 (0.09)	5.85 (0.09)	3.21 (0.07)	3.20 (0.09)	0.83 (0.00)	0.83 (0.00)	23.72 (7.31)	28.95 (11.1)
	P05	18.90 (2.14)	0.66 (0.05)	5.39 (0.08)	5.39 (0.08)	2.98 (0.07)	2.97 (0.08)	0.81 (0.00)	0.81 (0.00)	18.32 (5.33)	22.36 (9.01)
	P95	27.94 (3.47)	1.18 (0.1)	6.37 (0.11)	6.36 (0.09)	3.46 (0.08)	3.44 (0.09)	0.85 (0.00)	0.85 (0.00)	30.16 (9.86)	36.96 (13.87)
M. *Torque* *Min.* (kg∙m)	P50	1.81 (0.12)	0.15 (0.00)	0.47 (0.02)	0.50 (0.04)	0.33 (0.00)	0.33 (0.00)	0.04 (0.00)	0.04 (0.00)	3.63 (0.51)	3.84 (0.57)
F. *Torque* *Max.* (kg∙m)	P50	15.92 (1.79)	0.62 (0.05)	5.14 (0.07)	5.16 (0.08)	2.81 (0.07)	2.81 (0.08)	0.7 (0.00)	0.7 (0.00)	16.03 (4.73)	19.3 (8.21)
	P05	13.49 (1.50)	0.45 (0.03)	4.76 (0.06)	4.78 (0.07)	2.62 (0.06)	2.62 (0.07)	0.68 (0.00)	0.68 (0.00)	12.27 (3.38)	14.78 (6.53)
	P95	19.09 (2.18)	0.84 (0.07)	5.58 (0.09)	5.59 (0.08)	3.03 (0.07)	3.02 (0.08)	0.72 (0.00)	0.72 (0.00)	20.67 (6.34)	25.54 (9.81)
F. *Torque* *Min.* (kg∙m)	P50	1.08 (0.07)	0.11 (0.00)	0.33 (0.01)	0.35 (0.03)	0.23 (0.00)	0.23 (0.00)	0.03 (0.00)	0.03 (0.00)	2.26 (0.33)	2.39 (0.35)
*AngSpeed* *Max.* (°/s)	-	51.0 (6.1)	222.7 (39.4)	240.2 (43.9)	254.5 (40.3)	219.3 (22.3)	247.7 (21.4)	217.1 (51.2)	230.5 (48.9)	133.7 (21.5)	132.9 (25.2)
*AngSpeed* *Min.* (°/s)	-	10.0 (1.3)	40.9 (4.8)	41.6 (3.8)	41.3 (4.5)	64.0 (4.4)	64.5 (6.7)	66.2 (3.8)	67.7 (4.6)	40.4 (6.7)	40.2 (6.5)

Notes: R: Right side, L: left side, M: Male, F: Female, P: Percentile.

**Table 7:** Maximum and minimum forces for the 50th percentile; coefficients for 5th and 95th percentiles.

	Male				Female			
Joints	Min. (kg)	Max. (kg)	CoefP05	CoefP95	Min. (kg)	Max. (kg)	CoefP05	CoefP95
Lumbar	44.6	65.1	0.87	1.16	27.7	46.9	0.90	1.13
Cervical	6.8	8.8	0.82	1.20	5.5	7.1	0.83	1.21
Shoulders	4.2	13.6	0.96	1.05	3.1	12.7	0.96	1.04
Elbows	1.9	11.6	0.98	1.02	1.4	11.2	0.98	1.02
Wrists	0.5	10.6	0.99	1.01	0.4	10.5	0.99	1.01
Knees	36.9	80.6	0.83	1.18	27.2	60.4	0.84	1.18

**Table 8:** Maximum and minimum torque for the 50th percentile; coefficients for 5th and 95th percentiles.

	Male				Female			
Joints	Min. (kg∙m)	Max. (kg∙m)	CoefP05	CoefP95	Min. (kg∙m)	Max. (kg∙m)	CoefP05	CoefP95
Lumbar	1.81	22.95	0.82	1.22	1.08	15.92	0.85	1.20
Cervical	0.15	0.90	0.73	1.32	0.11	0.62	0.72	1.35
Shoulders	0.49	5.85	0.92	1.09	0.34	5.15	0.93	1.09
Elbows	0.33	3.20	0.93	1.08	0.23	2.81	0.93	1.08
Wrists	0.04	0.83	0.97	1.03	0.03	0.70	0.97	1.03
Knees	3.73	26.34	0.86	1.41	2.32	17.66	0.84	1.44

**Table 9:** Maximum and minimum angular velocities and accelerations.

	*AngularSpeed* (°/s)		*AngularAcceleration* (°/s^2^)	
Joints	Slow	Maximum	Slow	Maximum
Lumbar	10	51	50	255
Cervical	41	223	205	1115
Shoulder	53	247	265	1235
Elbow	54	233	270	1165
Wrist	66	224	330	1120
Knee	40	133	200	665

**Table 10:** Results of the P003 workplace example: *RiskPerMinute* values.

	Lumbar	Cervical	Shoulder R	Elbow R	Wrist R	Knee R	Shoulder L	Elbow L	Wrist L	Knee L
P003_Medium	22.9%	22.6%	8.8%	16.2%	15.4%	20.5%	9.1%	26.9%	14.2%	14.7%

**Table 11:** Example of the combination of workstation assessments considering a specific weight assigned to each situation.

	Weight	Lumbar	Cervical	Shoulder R	Elbow R	Wrist R	Knee R	Shoulder L	Elbow L	Wrist L	Knee L
P003_Medium	42.8%	22.9%	22.6%	8.8%	16.2%	15.4%	20.5%	9.1%	26.9%	14.2%	14.7%
P003_Low	28.6%	34.2%	24.1%	8.6%	21.7%	14.9%	19.6%	8.6%	27.3%	12.4%	24.7%
P003_Top	28.6%	21.8%	20.7%	14.1%	24.0%	16.3%	15.2%	12.8%	29.7%	13.1%	18.2%
P003_COMBI		25.8%	22.5%	10.3%	20.0%	15.5%	18.7%	10.0%	27.8%	13.4%	18.6%

**Table 12:** Example of a risk map and job rotation, considering a specific weight assigned to each situation (for combinations) or each workstation (for job rotation).

	Weight	Lumbar	Cervical	Shoulder R	Elbow R	Wrist R	Knee R	Shoulder L	Elbow L	Wrist L	Knee L
P001	20.0%	6.8%	25.4%	4.6%	11.4%	5.0%	4.5%	4.2%	10.3%	7.3%	4.7%
P002	20.0%	18.2%	16.7%	7.9%	15.7%	5.9%	5.3%	11.7%	18.7%	8.1%	4.3%
P003_COMBI	20.0%	25.8%	22.5%	10.3%	20.0%	15.5%	18.7%	10.0%	27.8%	13.4%	18.6%
P004	20.0%	13.6%	44.4%	22.4%	40.8%	36.4%	17.2%	24.4%	35.7%	37.4%	6.1%
P005	20.0%	30.4%	12.6%	7.9%	8.9%	4.9%	0.0%	9.4%	10.8%	8.4%	0.4%
Rotation		19.0%	24.3%	10.6%	19.4%	13.5%	9.1%	11.9%	20.7%	14.9%	6.8%

## Data Availability

Not applicable.

## Supplementary Materials

The following are available online at https://www.mdpi.com/article/10.3390/s21155139/s1, Table S1: Forces_Tables.xlsx.
